# Extraskeletal Chondroma of the Preauricular Region: A Case Report and Literature Review

**DOI:** 10.1155/2012/121743

**Published:** 2012-07-10

**Authors:** Futoshi Watanabe, Tadahiko Saiki, Yoshihisa Ochochi

**Affiliations:** Department of Otolaryngology, Head and Neck Surgery, Steel Memorial Hirohata Hospital, 3-chome, Yumesaki-cho, Hirohata-ku, Himeji 671-1122, Japan

## Abstract

An extraskeletal chondroma is a rare benign cartilaginous tumor that develops in soft tissue. Histologically, it is a lobulated nodule surrounded by a fibrous capsule; the inside consists of mature hyaline cartilage containing a few normal chondrocytes. We present a rare case of extraskeletal chondroma in the preauricular region. A 43-year-old man presented with a 2-cm-diameter right preauricular tumor that had been developing for 1 year. Magnetic resonance imaging showed a solid lobulated tumor in the right preauricular region, which was proximate to the capsule of the right temporomandibular joint (TMJ). This was subsequently resected under general anesthesia. The tumor was not in contact with the TMJ capsule and had not invaded the surrounding tissue, facilitating *en bloc* excision. Histopathologically, the tumor comprised mainly of hyaline cartilage containing chondrocytes with chondrocytic lacunae and was diagnosed as a chondroma. The postoperative period was uneventful, and there was no evidence of recurrence at the 2-year followup. We describe the clinical characteristics of our case and review the literature, emphasizing the differential diagnosis.

## 1. Introduction

Extraskeletal or soft-tissue chondromas are rare benign cartilaginous tumors that develop in soft tissues without bone or joint involvement, occurring predominantly in the hands and feet of adults in their third and fourth decades of life [1, 2]. In the head and neck, extraskeletal chondromas are rare, having been described mainly in the tongue [3, 4]. To our knowledge, only one extraskeletal chondroma has been reported in the preauricular region [5]. This paper describes an extremely rare extraskeletal chondroma that developed in the preauricular region of a 43-year-old man.

## 2. Case Presentation

A 43-year-old man was referred to our department complaining of a mass in the right preauricular region, which had been developing painlessly over 1 year. The physical examination revealed a hard mass approximately 2 cm diameter in the right preauricular region. Otoscopy showed swelling of the anterior wall in the right external acoustic meatus, covered by normal skin. There was no temporomandibular joint (TMJ) pain, trismus, or hearing loss, and there was no history of facial trauma. The general physical examination did not disclose any abnormalities.

**Figure 1:**
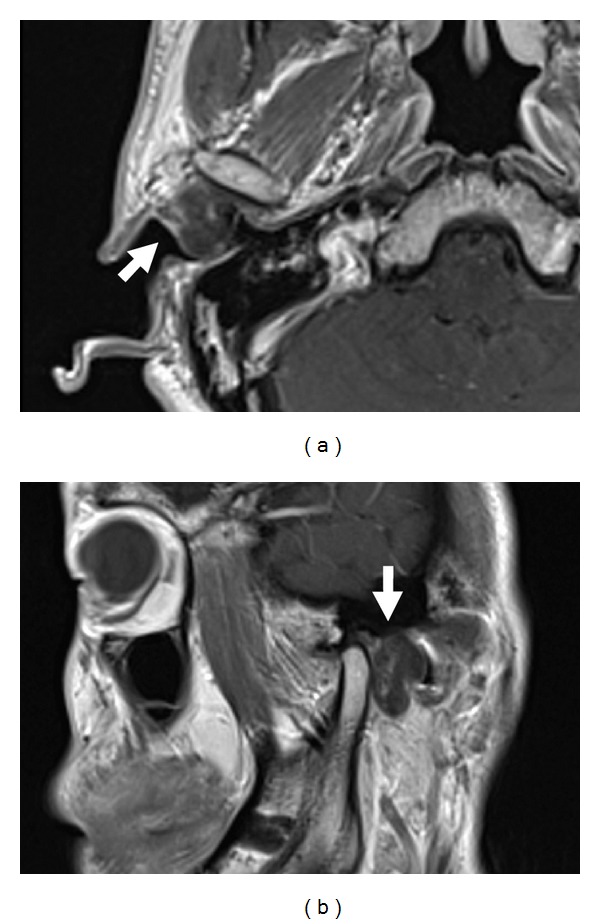
T1 gadolinium-enhanced axial (a) and sagittal (b) magnetic resonance images show a solid tumor (arrow) proximate to the capsule of the right temporomandibular joint in the right preauricular region.

Magnetic resonance imaging (MRI) revealed a solid lobulated lesion in the right preauricular region, which was proximate to the capsule of the right TMJ (Figure 1). The lesion was isointense relative to muscle on T1-weighted images, hyperintense on T2-weighted images, and partially enhanced after gadolinium injection.

**Figure 2:**
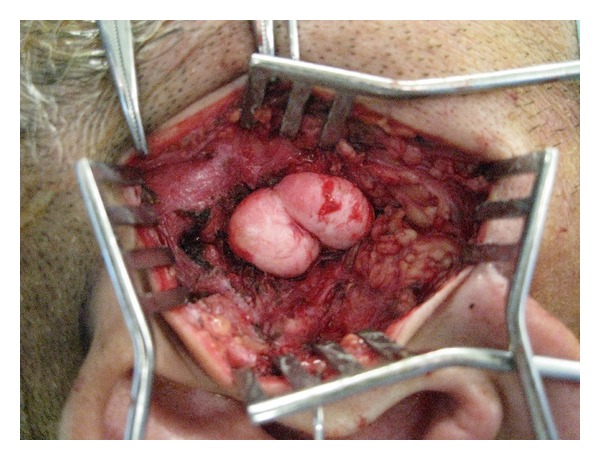
Intraoperative image shows *en bloc* excision of the tumor.

A benign tumor in the right TMJ was suspected clinically. The lesion was removed surgically via a preauricular incision under general anesthesia to determine the histopathological diagnosis. The lesion was independent of the parotid gland and was not in contact with either the TMJ or the cartilage of the acoustic meatus (Figure 2). Although the lesion was attached to the surface of the temporal bone, there was no invasion of the temporal bone, and the lesion was removed easily by *en bloc *excision.

**Figure 3:**
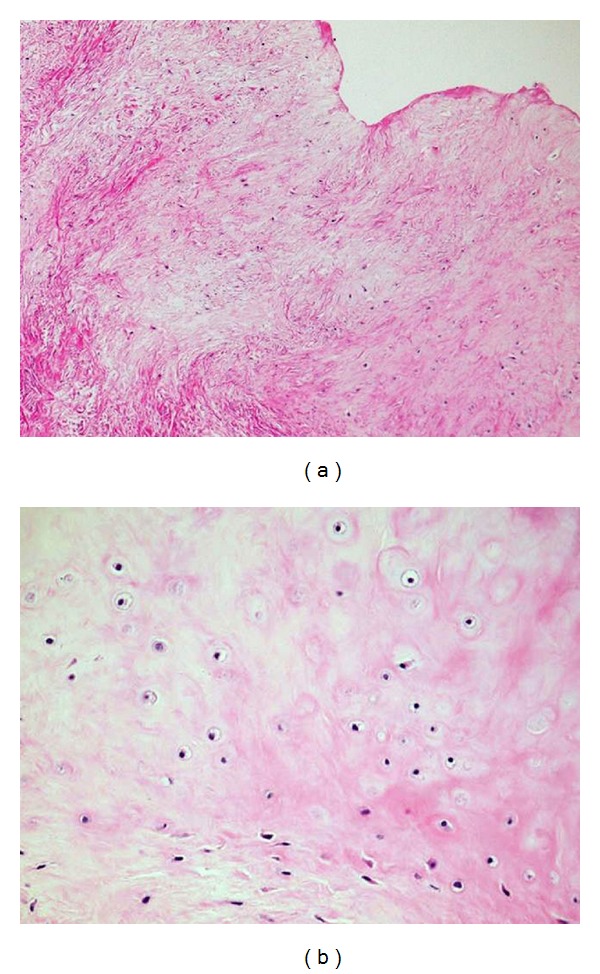
Histopathologically, (a) the tumor consists of hyaline cartilage and collagenous fibrous tissue (H&E stain, ×40). (b) The hyaline cartilaginous tissue contains homogeneous chondrocytes with round nuclei and chondrocytic lacunae (H&E stain, ×200).

The specimen was a 2.5 × 2 × 1.5 cm, well-circumscribed, nodular, white-grey, hard, elastic mass. Histopathologically, the tumor was well circumscribed and encapsulated by thin fibrous connective tissue. The tumor comprised mainly of hyaline cartilage, which contained chondrocyte with chondrocytic lacunae, with collagenous fibrous tissue in the center of lesion (Figure 3). The tumor had low cellularity, with no evidence of atypia or mitotic figures. Immunohistochemically, the chondrocytes expressed S-100 protein and vimentin, but were negative for epithelial cell markers. These histopathological findings revealed the diagnosis of chondroma.

The postoperative period was uneventful, and there was no evidence of recurrence at the 2-year followup.

## 3. Discussion

Extraskeletal or soft-tissue chondromas are rare benign cartilaginous tumors that occur in tissue unrelated to bone and are usually found in close proximity to tendons or joint capsules [1, 2]. These tumors arise most commonly in the hands and feet of middle-aged adults and have an equal sex distribution. The clinical symptoms are usually related to those of a slowly, insidiously expanding mass that occasionally causes tenderness or pain. Less common locations include the chest and abdominal wall, lung, fallopian tube, and other visceral organs. In the head and neck, extraskeletal chondromas are most prevalent in the tongue [3, 4] and rare in the cheek [6, 7], parotid gland [8, 9], neck [10], masticatory space [11], parapharyngeal space [12], and masseter muscle [13]. Our literature survey found only one previous account of a chondroma in the preauricular region [5].

The literature review of extraskeletal chondromas of the parotid region is summarized in Table 1. The five cases of parotid chondroma, including ours, included two in the parotid gland, two in the preauricular region, and one in the masseter muscle. Patients were three males and two females. The median patient age was 45.0 (range 32–54) years. The greatest diameter of the tumor was reported in four cases and averaged 3.2 (range 2.5–4.0) cm. Wide local excisions were performed in all cases, and no recurrence was mentioned for cases for which follow-up data were available.

The etiology of extraskeletal chondroma is uncertain. In the literature on tongue chondromas, it is felt that either these lesions develop from residual embryonal tissue in an area of preexisting fetal cartilage or pluripotent mesenchymal cells undergo metaplasia, differentiating into cartilage as a result of some irritating stimulus [3]. However, the exact causal relationship is poorly defined.

Histologically, the lesion is a lobulated nodule surrounded by a fibrous capsule filled with mature hyalinized cartilage tissue, with no association with underlying bone. Locally, it may be associated with secondary degeneration within the matrix, such as cystic change, ossification, calcification, fibrosis, and mucinous change [1, 2].

Radiologically, extraskeletal chondromas are well-demarcated extraskeletal masses with a periarticular location. Computed tomography (CT) shows an iso- or hyperdense soft-tissue mass without underlying bone involvement; calcifications are seen in 33–70% of cases [2]. MRI shows a sharply delineated lobulated mass with hypo- to isointense signals relative to muscle on T1-weighted images, hyperintense signals on T2-weighted images, and various patterns of enhancement with contrast medium [14]. In our case, no calcifications were observed in the histological specimens, although no preoperative CT was obtained.

For lesions in the preauricular region, the differential diagnosis includes parotid gland and TMJ tumors, such as osteochondromas and synovial chondromatosis. The chondromatosis change of a pleomorphic adenoma or mixed parotid tumor can be similar to that of a chondroid tumor [15]. An osteochondroma is a cartilage-capped bony projection on the external surface of bone; these usually occur in younger patients and stop developing after adolescence. Histologically, synovial chondromatosis can be similar to an extraskeletal chondroma, but there are multiple nodules arising in the synovium; they are usually intra-articular and can involve bone. In our case, the preoperative diagnosis was a TMJ tumor because the lesion was proximate to the TMJ capsule, although it was not in contact with the TMJ, and there was no evidence of surrounding tissue invasion. Histopathologically, the lesion was mainly hyaline cartilage and fibrous tissue without severe atypia or mitosis and was diagnosed as a chondroma. Immunohistochemistry is useful because the tumor cells are positive for S-100 protein and vimentin and negative for epithelial and myoepithelial cell markers [8, 9].

It is also important to distinguish chondromas from malignant cartilaginous neoplasms; however, it may be difficult to distinguish between a chondroma and low-grade chondrosarcoma histologically. Some clinical features should raise a suspicion of malignancy, including older age at presentation, rapid growth, and the invasion of surrounding structures [2]. Recurrence or metastasis also helps with the differential diagnosis.

Wide local excision is recommended for treatment owing to the 10–15% recurrence rate [1, 2]. In our case, the tumor was well encapsulated and excised completely, and there was no evidence of recurrence 2 years postoperatively. We plan to observe the patient for the long term because of the possibility of late tumor recurrence.

In conclusion, we present a very rare extraskeletal chondroma of the preauricular region and review the literature on extraskeletal chondromas of the parotid region. 

## Figures and Tables

**Table 1:** The literature review of extraskeletal chondromas of the parotid region.

Author (year)	Age	Sex	Duration	Location	Size (cm)	Treatment	Recurrence
Kostpoloulos et al. (1993) [9]	32	M	7 years	Parotid gland	NA	Excision	—
Aslam and Haqqani (2006) [8]	47	F	2 years	Parotid gland	4 × 3 × 2	Excision	—
Vazques Maha et al. (2007) [5]	54	F	4 years	Preauricular region	2.5 × 2.5	Excision	—
Falleti et al. (2009) [13]	49	M	6 years	Masseter muscle	4	Excision	—
Present case	43	M	1 year	Preauricular region	2.5 × 2 × 1.5	Excision	—

TMJ: temporomandibular joint; NA: not available.

## References

[1] Chung EB, Enzinger FM (1978). Chondroma of soft parts. *Cancer*.

[2] Kransdorf MJ, Meis JM (1993). From the archives of the AFIP. Extraskeletal osseous and cartilaginous tumors of the extremities. *Radiographics*.

[3] Yasuoka T, Handa Y, Watanabe F, Oka N (1984). Chondroma of the tongue. Report of a case. *Journal of Maxillofacial Surgery*.

[4] Sera H, Shimoda T, Ozeki S, Honda T (2005). A case of chondroma of the tongue. *International Journal of Oral and Maxillofacial Surgery*.

[5] Vázquez Mahía I, López-Cedrún Cembranos JL, Ferreras Granado J, Lorenzo Franco F (2007). Temporomandibular juxtaarticular chondroma: case report. *Medicina Oral, Patología Oral y Cirugía Bucal*.

[6] Onodera K, Xu H, Kimizuka S, Echigo S, Ooya K (2005). Chondroma of the cheek: a case report. *International Journal of Oral and Maxillofacial Surgery*.

[7] Blum MR, Danford M, Speight PM (1993). Soft tissue chondroma of the cheek. *Journal of Oral Pathology and Medicine*.

[8] Aslam MB, Haqqani MT (2006). Extraskeletal chondroma of parotid gland. *Histopathology*.

[9] Kostpoloulos IS, Daniilidis I, Velegrakis G, Papadimitriou CS (1993). Chondroma of parotid gland. Clinical, histological and immunohistochemical findings in a unique case. *Laryngo- Rhino- Otologie*.

[10] Kamysz JW, Zawin JK, Gonzalez-Crussi F (1996). Soft tissue chondroma of the neck: a case report and review of the literature. *Pediatric Radiology*.

[11] De Riu G, Meloni SM, Gobbi R, Contini M, Tullio A (2007). Soft-tissue chondroma of the masticatory space. *International Journal of Oral and Maxillofacial Surgery*.

[12] Wang DH, Guan XL, Xiao LF, Zhang XP, Chen MG, Sun KM (1998). Soft tissue chondroma of the parapharyngeal space: a case report. *The Journal of Laryngology & Otology*.

[13] Falleti J, De Cecio R, Mentone A (2009). Extraskeletal chondroma of the masseter muscle: a case report with review of the literature. *International Journal of Oral and Maxillofacial Surgery*.

[14] Woertler K, Blasius S, Brinkschmidt C, Hillmann A, Link TM, Heindel W (2001). Periosteal chondroma: mr characteristics. *Journal of Computer Assisted Tomography*.

[15] Pogrel MA (1979). Tumours of the salivary glands: a histological and clinical review. *British Journal of Oral Surgery*.

